# Factors Influencing the Sharing of Personal Health Data Based on the Integrated Theory of Privacy Calculus and Theory of Planned Behaviors Framework: Results of a Cross-Sectional Study of Chinese Patients in the Yangtze River Delta

**DOI:** 10.2196/46562

**Published:** 2023-07-06

**Authors:** Jingjin Shi, Rui Yuan, Xueming Yan, Miao Wang, Jun Qiu, Xinhua Ji, Guangjun Yu

**Affiliations:** 1 International Peace Maternity and Child Health Hospital School of Medicine Shanghai Jiao Tong University Shanghai China; 2 Miaohang Town Community Health Service Center, Baoshan District Shanghai China; 3 Shanghai Children’s Hospital Shanghai Jiao Tong University Shanghai China; 4 School of Medicine The Chinese University of HongKong Shenzhen China

**Keywords:** personal health data, data sharing, influencing factor, privacy calculus, Theory of Planned Behavior

## Abstract

**Background:**

The health care system in China is fragmented, and the distribution of high-quality resources remains uneven and irrational. Information sharing is essential to the development of an integrated health care system and maximizing its benefits. Nevertheless, data sharing raises concerns regarding the privacy and confidentiality of personal health information, which affect the willingness of patients to share information.

**Objective:**

This study aims to investigate patients’ willingness to share personal health data at different levels of maternal and child specialized hospitals in China, to propose and test a conceptual model to identify key influencing factors, and to provide countermeasures and suggestions to improve the level of data sharing.

**Methods:**

A research framework based on the Theory of Privacy Calculus and the Theory of Planned Behavior was developed and empirically tested through a cross-sectional field survey from September 2022 to October 2022 in the Yangtze River Delta region, China. A 33-item measurement instrument was developed. Descriptive statistics, chi-square tests, and logistic regression analyses were conducted to characterize the willingness of sharing personal health data and differences by sociodemographic factors. Structural equation modeling was used to assess the reliability and validity of the measurement as well as to test the research hypotheses. The STROBE (Strengthening the Reporting of Observational Studies in Epidemiology) checklist for cross-sectional studies was applied for reporting results.

**Results:**

The empirical framework had a good fit with the chi-square/degree of freedom (*χ*^2^/*df*)=2.637, root-mean-square residual=0.032, root-mean-square error of approximation=0.048, goodness-of-fit index=0.950, and normed fit index=0.955. A total of 2060 completed questionnaires were received (response rate: 2060/2400, 85.83%). Moral motive (β=.803, *P*<.001), perceived benefit (β=.123, *P*=.04), and perceived effectiveness of government regulation (β=.110, *P*=.001) had a significantly positive association with sharing willingness, while perceived risk (β=–.143, *P*<.001) had a significant negative impact, with moral motive having the greatest impact. The estimated model explained 90.5% of the variance in sharing willingness.

**Conclusions:**

This study contributes to the literature on personal health data sharing by integrating the Theory of Privacy Calculus and the Theory of Planned Behavior. Most Chinese patients are willing to share their personal health data, which is primarily motivated by moral concerns to improve public health and assist in the diagnosis and treatment of illnesses. Patients with no prior experience with personal information disclosure and those who have tertiary hospital visits were more likely to share their health data. Practical guidelines are provided to health policy makers and health care practitioners to encourage patients to share their personal health information.

## Introduction

### Background

With the rapid development of medical information, health care data are becoming an increasingly valuable and fundamental strategic resource for countries around the world. Data sharing connects and integrates medical information systems and public health data. This is conducive to improving the efficiency of medical services, increasing the value of scientific research, verifying research results, promoting advances in discoveries, and realizing personalized health management and monitoring. As part of the effort to promote the rapid development of health care, data producers are required to provide health care data for scientific research and clinical practice. Health care data involve sensitive information such as laboratory data, family history, medical history, and medication history. As the public becomes more aware of privacy and security, their willingness to share health care data must be addressed. In China, health care data are in the initial stage of development, and medical institutions control the majority of patients’ health care data, which are highly valuable for exploitation. Unsound laws and regulations, lack of specific health information standards, inappropriate management and publicity, privacy leakage, and information security risks lead to low willingness of the public to share. Despite this, there are few studies that explored patient perspectives on the willingness to share personal health data in China. In this study, we will investigate patients’ willingness to share personal health data and identify their influencing factors using a Theory of Privacy Calculus and Theory of Planned Behavior research framework. This will serve as a reference for the early and orderly implementation of medical data sharing.

### Theoretical Model and Research Hypothesis

#### Theory of Privacy Calculus

The Theory of Privacy Calculus is considered to be the most practical theory for analyzing the personal privacy and security of information system users. Initially, it was applied to electronic commerce, but now it is flourishing in the field of information systems. It is widely used by scholars in various countries for privacy disclosure or information sharing research [1]. The Theory of Privacy Calculus is a typical behavioral model extension, and this study considers data sharing as a cost-benefit analysis behavior that explores individuals’ attitudes and behaviors when facing privacy and security issues [2]. The theory argues that individuals ultimately decide whether to disclose personal information by weighing the relationship between the perceived benefits and the perceived risks. If the perceived benefits of sharing personal information are greater than the perceived risks, then data sharing behavior will be rationally achieved at the expense of partial privacy [3]; however, if the perceived benefits are less than the perceived risks, services will be suspended to avoid the risk of personal privacy leakage. The theory focuses not only on the enablers of information sharing, but also on the impediments caused by privacy leakage. This has been widely used in different contexts to explain the willingness to share personal data. In previous studies in areas such as e-commerce [4], social networks [5], internet of things [6], and wearable devices [7], it has been demonstrated that the perception of benefits can have a significant positive impact on the willingness to provide data. Perceived benefits include trust, perceived ease of use, perceived usefulness, perceived value, personalization, social rewards, financial rewards, and altruistic motives [8]. The balance between privacy protection and economic or social benefit most strongly influenced public preference for sharing personal health data [9]. Patients acknowledge that sharing personal health data has a number of potential benefits, but there are also potential risks associated with it. This is because health care data are subject to more personal privacy than other general information [10]. People are reluctant to provide personal information when the risks are perceived to be high. Numerous studies in the fields of e-commerce [11], social networks [12], location-based services [13], and scientific data sharing [14] have confirmed a significant negative relationship between perception of risk and willingness to share. However, fewer scholars have conducted empirical studies on the willingness to share individual health care data based on the Theory of Privacy Calculus [7].

#### Theory of Planned Behavior

The Theory of Planned Behavior is one of the most widely used psychosocial models for predicting and explaining intentions and behaviors. It assumes that a person’s behavior is a nonpurely rational behavior that is difficult to predict, and that individual behavior intentions are influenced by attitudes, subjective norms, and external circumstances. The more positive the attitude, the stronger the subject norms and perceived behavioral control and the stronger the willingness to perform a specific behavior [15]. The Theory of Planned Behavior has been widely used in behavioral research on new technologies and information systems. Hence, this study argues that it is also applicable to the study of willingness to share data in health care.

Subjective norm refers to external social pressures felt by an individual when considering whether to perform a given behavior, and it reflects the effect of the significant others or groups on that individual’s behavior. It has been demonstrated that subjective norm is one of the influencing factors that promote data sharing and positively affect users’ willingness to use new information systems [16]. Eisenberger et al [17] proposed the concept of social reward based on subjective norms, which is the material reward or spiritual fulfillment that an individual receives after performing a specific behavior. Previous research has found that the societal benefits of data sharing outweigh considerations of individual privacy and security [16]. Once some patients realize that sharing personal health data can contribute to advancing medicine, they will increase their willingness to share their data and achieve the interconnection of medical information [18].

Perceived behavioral control is defined as a person’s perception of being able to control their behavior. Previous research has found that increasing information control can reduce information system users’ concerns about privacy breaches. When patients are clear about who controls personal information and how it is used, it will weaken perceived risks and increase perceived benefits, which in turn will ultimately increase their willingness to share [19]; otherwise patients’ willingness to share will decrease [20].

Social reward is defined as the financial gain and spiritual fulfillment that an individual receives after performing a specific behavior. Monetary benefit is one type of social reward, and may have a significant impact on willingness to share. This is because users are willing to take possible risks and receive explicit compensation or benefits. Accordingly, perceived risk is negatively related to monetary benefit, whereas monetary benefit is positively related to willingness to share. In scientific research situations, researchers frequently compensate patients who agree to participate in research and disclose their health information. This is necessary to attract and recruit participants.

Moreover, trust has been shown to be an important factor in promoting cross-institutional information sharing and in predicting individual willingness to share [21]. As the online environment is uncertain and high risk, most people lack trust in the flow of data and the purpose for which they are accessed. They may be reluctant to share their personal information with third parties due to a variety of reasons, including the loss of competitive advantage, unequal quality of data, or the misuse of information by others. It has been demonstrated that trust can significantly increase users’ perceptions of benefits and decrease perceived risks in the use of novel technologies [16]. Consequently, patients are more likely to share personal information with organizations whose information systems are secure and stable.

The concept of privacy concern can be defined as the subjective perception of the protection of personal information by a user. Patients have expressed concerns that the use of information systems may result in a breach of their privacy as a result of collection, unauthorized access, or misappropriation of personal information [22]. Previous studies have confirmed that privacy concerns have a positive effect on perceived risk and have a negative effect on willingness to share [23,24].

In addition to individual influencing factors, policies and regulations can be effective in reducing risks and facilitating cross-organizational data sharing. In the current turbulent environment in which industry regulation is still imperfect and hospitals are unable to fully ensure the security of patient information and provide adequate privacy protection, government regulations can provide safeguards for patients who are subjected to privacy violations [25]. Accordingly, we propose the hypothesis that the perceived effectiveness of government regulation will reduce patients’ perceived risk and increase their willingness to share their personal health information.

**Figure 1:**
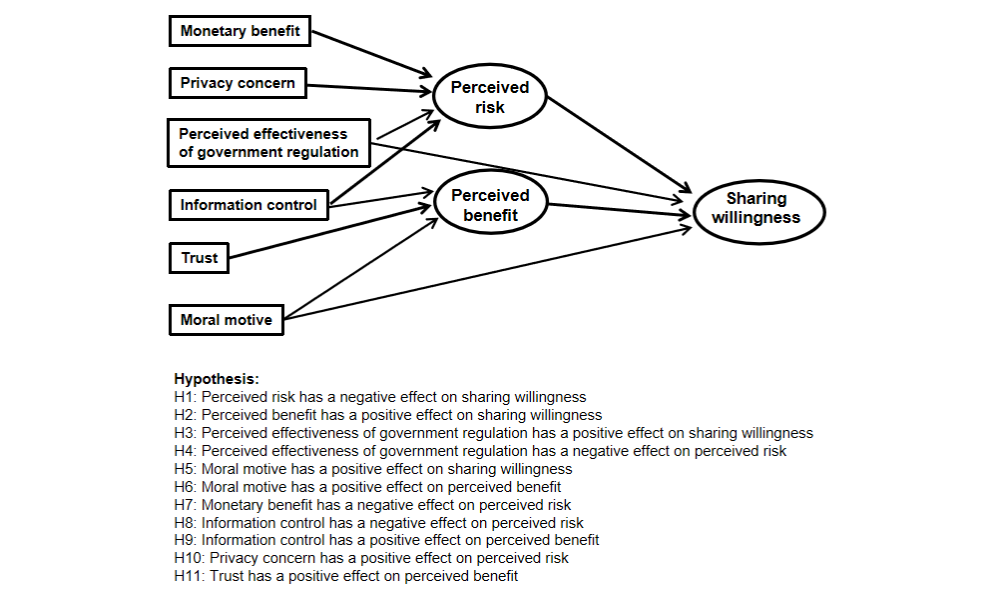
The proposed research model.

In summary, this paper integrates the Theory of Privacy Calculus and the Theory of Planned Behavior for explaining the willingness to share personal health data. It also measures the influencing factors of patient groups based on 2 latent variables, namely, perceived risk and perceived benefit. The latent variables privacy concern, monetary benefit, and perceived effectiveness of government regulation are used to assess perceived risk, while the latent variables information control, moral motive, and trust are used to measure perceived benefit. The damage caused by a breach of medical data will be difficult to quantify and estimate because medical data are more valuable and highly sensitive than other types of data. By constructing a theoretical model and validating it with empirical data, we aim to elucidate the mechanisms underlying the willingness of Chinese patients to share personal health data. Figure 1 illustrates the research framework and hypotheses proposed based on the aforesaid rationale and results of previous research.

## Methods

### Study Design, Setting, and Participants

This is a cross-sectional field study to investigate patients’ willingness to share personal health data. A convenience sampling method was used to select 5 maternal and child health hospitals in the Yangtze River Delta, China as sample hospitals. The paper questionnaires were answered anonymously and empirical data were collected on the spot from September 1 to October 30, 2022. The inclusion criteria for participants were as follows: (1) ability to fill out the questionnaire independently, with clear consciousness and no obvious cognitive impairment; (2) willingness to voluntarily participate in this study; and (3) age range of 18-60 years. The exclusion criteria were as follows: (1) having a mental disorder that prevented normal communication and (2) refusing to participate in this investigation. This cross-sectional field survey adheres to the STROBE (Strengthening the Reporting of Observational Studies in Epidemiology) statement [26,27]. The guidelines provide a 22-item checklist outlining standards (eg, title, abstract, introduction, methods, results, discussion, and funding) to ensure that observational studies are systematically conducted and reported (Multimedia Appendix 1).

### Sample Size

Considering the sample balance and dropout, a minimum sample size of 1600 should be used for this study to obtain adequate statistical power by collecting 800 questionnaires from the tertiary hospital and 200 questionnaires from each of the 4 secondary hospitals. As this is a cross-sectional study, the sample size was calculated using the following formula [28]:



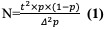



According to the above formula for the sample size of the cross-sectional study, where t=1.96 and α=.05, *P*=.50 was assumed because there is currently no reliable estimate of the percentage of patients in China willing to share health information, as the overall variance is the greatest at this time. Substituting these values into the equation yields an initial sample size of N=1.96 × 1.96 × 0.5 × (1–0.5)/(0.05 × 0.05)=384, which when combined with a response rate of approximately 50% from relevant studies yields the sample size in each hospital to be 384/0.5=768. Further, based on the sample size requirements for exploratory factor analysis and structural equation modeling statistical analysis, it is recommended that the sample size is 5-10 times greater than the number of measurements items, and should not be less than 150, and the larger the sample, the better [29]. Thus, the recommended sample size of the 33-item scale is 330, which is also in line with the sample size calculated above.

### Variable, Data Sources, and Measures

The questionnaire included 2 parts—demographic characteristics and multiple-item scales. Demographic variables assessed in this study were gender, age, educational level, hospital level, household income, place of residence, perceived health status, online medical service usage (years), and experience. In the proposed model, 9 constructs were based on the Theory of Planned Behavior and the Theory of Privacy Calculus, which were adapted from validated scales and had wording modifications to accommodate individual behavioral intentions in the health care context. In Table 1, definitions and sources are presented for each construct based on prior research. A team of 5 experts in the field of health informatics and health information exchange (HIE) was invited to review the initial questionnaire. A content validity index test was conducted to verify its accuracy, completeness, readability, and formatting based on the feedback and suggestions of the experts. Every item was evaluated by each expert on a scale to determine whether it was congruent (or relevant) to the construct. Hence, we calculated the percentage of items that each expert deemed relevant, and the average of these percentages was computed. The average congruency percentage was 94%, exceeding 90% [30]. As a result, the average congruency percentage was considered acceptable for use in this study. To avoid misunderstandings related to wording, a professional translator translated the questionnaire from English to Chinese, followed by a back-translation [31].

Prior to conducting the main study, we conducted a pilot survey with 109 patients at a tertiary specialized hospital in Shanghai to ensure the reliability and validity of the instrument. In response to the participants’ suggestions, we removed the words marked as ambiguous and modified the questions to ensure they are clear and easy to understand. Taking into account the results of the pretest, including Cronbach α coefficient, corrected-item total correlation (CITC) values, and communality values, the questionnaire was modified by removing 3 items from the “Informed Consent” scale. The final measurement instruments used in this study are presented in Multimedia Appendix 2. To illustrate the robustness of the framework, we report a series of surveys performed using the framework to measure the effect of the following 9 factors on willingness of sharing data: perceived benefit, perceived risk, perceived effectiveness of government regulation, monetary benefit, moral motive, information control, privacy concern, trust, and demographics. Participants reported their responses on a 5-point Likert scale ranging from 1 (completely disagree) to 5 (completely agree).

**Table 1:** Definitions of constructs.

Construct	Definition	References
Perceived risk	The potential costs or losses associated with sharing personal health care data.	[12]
Perceived benefit	The benefits or positive outcomes that may result from sharing personal health care data.	[12]
Perceived effectiveness of government regulation	The level of confidence in the accuracy, reliability, and comprehensiveness of laws and regulations regarding privacy protection and information security practices.	[29]
Monetary benefit	The degree of remuneration and material satisfaction that sharing personal health data can bring, such as subsidies.	[30]
Moral motive	The level of respect and spiritual fulfillment that sharing personal health data can bring.	[30]
Information control	The self-perception of being in control or control when sharing personal health data.	[31,32]
Privacy concern	The concerns about potential privacy breaches due to sharing of one’s health data	[2,33]
Trust	The willingness to assume the risks and harms that may result from the sharing of personal health data because of a belief that the person with whom the data are shared will take protective measures.	[23,34]
Sharing willingness	The subjective possibility of sharing or planning to share personal health care data.	[8]

### Data Quality Control and Bias

The investigators were provided with a researcher’s manual and received homogeneous training. This survey was conducted in person by 2 postgraduates from Shanghai Jiao Tong University School of Public Health to assist participants in understanding the meaning of questions and ensure the completion of collected questionnaires. EpiData 3.1 (EpiData Association) software was used to create a database; 2 investigators were responsible for double entry and logical verification. To ensure the validity of the questionnaire, the researcher predetermined the criteria for judging invalid questionnaires: (1) The questionnaire included a few general knowledge questions, which would be invalid if the responses were incorrect; (2) all the answers in the entire questionnaire were the same; (3) logical consistency was checked for inconsistencies. After eliminating incomplete and invalid questionnaires, a sample of 2060 valid questionnaires was finally collected. Thus, the actual number of questionnaires collected in this study was much larger than the minimum sample size required for factor analysis and structural equation modeling analysis, that is, the survey sample size should be 5-10 times larger than the number of measurement items (33 items). In light of this, we were confident that the final study results will not be biased by the deletion of some invalid questionnaires.

### Statistical Analysis

Descriptive statistics, frequencies, percentages of collected data, chi-square statistics, and logistic regression were conducted using SPSS Statistics, version 24.0 (IBM Corp). Path analysis was performed in SPSS Amos, version 24.0 (IBM Corp) to validate the research model and test the research hypotheses. A chi-square test was used to analyze the sharing willingness of all control variables. To determine participants’ intention to share their personal health data, we conducted a stepwise logistic regression analysis based on their demographics and health-related characteristics. The regression model only included independent variables significantly associated with the interest outcome in the univariate analysis. A 2-step approach was used for the structural equation modeling. The reliability and validity of the measurement model were examined in the first step, and the path analysis was tested in the second step. Significance was determined at a *P* value of <.05.

### Ethics Approval

This study was approved by the Ethics Review Committee, Shanghai Children’s Hospital, Shanghai Jiao Tong University (approval number 2021R077-E01). All respondents participated in this study voluntarily and anonymously on the basis of informed consent. Informed consent was obtained from all participants in the primary data collection, and the original informed consent allows the secondary analysis without additional consent.

## Results

### Sample Characteristics

In this study, patients from 5 member hospitals of a maternal and child specialty alliance were invited as survey respondents. The core hospital was a tertiary specialized hospital in Shanghai, while the member hospitals were 4 secondary maternal and child health hospitals randomly selected in the Yangtze River Delta (eg, Shanghai, Zhejiang, Jiangsu, and Anhui). A total of 2400 questionnaires were distributed, and 2060 questionnaires collected were valid (response rate: 85.83%). As shown in Table 2, female patients accounted for 78.40% (1615/2060) of the total, age range was mainly 31-40 years (972/2060, 47.18%), education was mainly undergraduate (918/2060, 44.56%), and self-assessed health status was good (1238/2060, 60.09%). In terms of hospital level, 45.92% (946/2060) of participants visited secondary hospitals and 54.07% (1114/2060) visited tertiary hospitals. Most respondents were not sure whether they had ever experienced personal health information leakage (1035/2060, 50.24%) and online medical services had not been used for a long time (even among this group, 825/2060, 40.04%, used these services for less than 1 year).

**Table 2:** Characteristics of the respondents (N=2060).

Characteristics			Value, n (%)
**Gender**			
	Male	445 (21.60)	
	Female	1615 (78.40)	
**Age** **(years)**			
	<21	48 (2.33)	
	21-30	754 (36.60)	
	31-40	972 (47.18)	
	41-50	216 (10.49)	
	51-60	53 (2.57)	
	>60	17 (0.83)	
**E** **ducation** **level**			
	High school or below	345 (16.75)	
	College	518 (25.15)	
	Bachelor	918 (44.56)	
	Master or above	279 (13.54)	
**Hospital level**			
	Secondary	946 (45.92)	
	Tertiary	1114 (54.08)	
**Household income (yuan^a^ per year)**			
	<100,000	519 (25.19)	
	100,000-199,999	581 (28.20)	
	200,000-299,999	370 (17.96)	
	300,000-399,999	201 (9.75)	
	400,000-499,999	138 (6.69)	
	>500,000	251 (12.18)	
**P** **lace** **of residence**			
	Shanghai	1295 (62.86)	
	Zhejiang	193 (9.36)	
	Jiangsu	211 (10.24)	
	Anhui	330 (16.01)	
	Others	31 (1.50)	
**Perceived h** **ealth status**			
	Poor	60 (2.91)	
	General	762 (36.99)	
	Good	1238 (60.10)	
**Online medical service usage years**			
	≤1	825 (40.05)	
	2-3	535 (25.97)	
	4-5	207 (10.05)	
	≥6	170 (8.25)	
	Never	323 (15.68)	
**Experiences of information leaks**			
	Yes	310 (15.05)	
	No	715 (34.71)	
	Unknown	1035 (50.24)	
**Experiences of sharing personal health data for scientific research**			
	Yes	174 (8.45)	
	No	1886 (91.55)	

^a^1 yuan=US $0.14.

### Control Variables

Several variables other than the core variables, which are not included in the proposed model of this study, nonetheless may affect the interrelationships between the constructs, and thus these have been controlled for. These control variables were gender, age, education level, department, household income, perceived health status, experiences of information leaks, experiences of sharing personal health data for scientific research, experiences of sharing personal health data for health care services, place of residence, and hospital level. Although the proposed model appears to represent patients’ intention and determine their willingness to share personal health information, the effects of control variables were not negligible. Based on the results of the questionnaire, the mean of the “willingness to share” was 3.496, the SD was 0.764, and the median was 3.50. Scores less than 3.50 were classified as the low willingness group, whereas scores higher than 3.50 were classified as the high willingness group. As a result, there was a difference in the willingness to share personal health data based on age (*χ*^2^=13.921, *P*=.02), perceived health status (*χ*^2^=10.943, *P*=.004), experiences of personal information leakage (*χ*^2^=19.713, *P*<.001), and hospital level (*χ*^2^=4.683, *P*=.03; Table 3).

Based on the 5 factors screened out from the univariate analysis as independent variables, a logistic regression forward stepwise method with entry level α=.05 and exclusion level β=.10 was used to construct a multivariate analysis regression model of willingness to share personal health data based on individual demographics (Table 4). The results of the Hosmer-Lemeshow goodness-of-fit test showed that *χ*^2^=12.426, *P*=.13, indicating that the model fit was good. Among the control variables, only hospital level (odds ratio 1.224; *P*=.03) and personal health information breach experience (odds ratio 0.806; *P*<.001) significantly affected willingness to share health information, meaning that patients without experience of personal information disclosure and tertiary hospital visits are more likely to share their personal health information with providers. However, there was no effect of age, gender, education level, income, perceived health status, or place of residence on willingness to share health information.

**Table 3:** The relationship between willingness to share personal health data and different demographic variables (N=2060).

Characteristics			Willingness to share				Chi-square		*P* value
			Low willingness, n/N (%)^a^		High willingness, n/N (%)				
**Gender**							1.240		.26
	Male	270/1203 (22.44)		175/857 (20.42)					
	Female	933/1203 (77.56)		682/857 (79.58)					
**Age** **(years)**							13.921		.02
	<21	34/1199 (2.84)		14/861 (1.63)					
	21-30	428/1199 (35.70)		326/861 (37.86)					
	31-40	582/1199 (48.54)		390/861 (45.30)					
	41-50	116/1199 (9.67)		100/861 (11.61)					
	51-60	25/1199 (2.09)		28/861 (3.25)					
	>60	14/1199 (1.17)		3/861 (0.35)					
**Education level**							6.078		.19
	High school or below	221/1203 (18.37)		127/857 (14.82)					
	College	306/1203 (25.44)		212/857 (24.74)					
	Bachelor	523/1203 (43.47)		392/857 (45.74)					
	Master	135/1203 (11.22)		112/857 (13.07)					
	Doctor	18/1203 (1.50)		14/857 (1.63)					
**Household income (yuan^b^ per year)**							6.900		.23
	<100,000	319/1201 (26.56)		197/859 (22.93)					
	100,000-199,999	326/1201 (27.14)		258/859 (30.03)					
	200,000-299,999	209/1201 (17.40)		161/859 (18.74)					
	300,000-399,999	111/1201 (9.24)		90/859 (10.48)					
	400,000-499,999	81/1201 (6.74)		57/859 (6.64)					
	>500,000	155/1201 (12.91)		96/859 (11.18)					
**Perceived health status**							10.943		.004
	Poor	30/1206 (2.49)		30/854 (3.51)					
	General	477/1206 (39.55)		285/854 (33.37)					
	Excellent	699/1206 (57.96)		539/854 (63.11)					
**Experiences of sharing personal health data for scientific research**							7.918		.02
	No	785/1200 (65.42)		543/860 (63.14)					
	Yes	84/1200 (7.00)		90/860 (10.47)					
	Unknown	331/1200 (27.58)		227/860 (26.40)					
**Experiences of information leaks**							19.713		<.001
	No	372/1203 (30.92)		343/857 (40.02)					
	Yes	201/1203 (16.71)		109/857 (12.72)					
	Unknown	630/1203 (52.37)		405/857 (47.26)					
**Experiences of sharing personal health data for health care services**							0.290		.59
	No	194/1203 (16.13)		131/857 (15.29)					
	Yes	1009/1203 (83.87)		726/857 (84.71)					
**P** **lace** **of residence**							0.429		.98
	Shanghai	745/1198 (62.19)		550/862 (63.81)					
	Zhejiang	114/1198 (9.52)		79/862 (9.16)					
	Jiangsu	124/1198 (10.35)		87/862 (10.09)					
	Anhui	196/1198 (16.36)		134/862 (15.55)					
	Others	19/1198 (1.59)		12/862 (1.39)					
**Hospital level**							4.683		.03
	Secondary	578/1204 (48.01)		371/856 (43.34)					
	Tertiary	626/1204 (51.99)		485/856 (56.66)					

^a^Based on the results of the questionnaire, the mean of the “willingness to share” was 3.496, the SD was 0.764, and the median was 3.50. Scores less than 3.50 were classified as the low willingness group, whereas scores higher than 3.50 were classified as the high willingness group.

^b^1 yuan=US $0.14.

**Table 4:** Logistic regression model: willingness to share personal health data (N=2060).

Variable	B	SE	Wald	*P* value	Odds ratio (95% CI)
Age	0.026	0.056	0.217	.64	1.026 (0.920-1.145)
Perceived health status	0.089	0.082	1.179	.28	1.093 (0.931-1.284)
Hospital level	0.202	0.092	4.794	.03	1.224 (1.021-1.467)
Experiences of information leaks	–0.216	0.054	16.134	<.001	0.806 (0.726-0.895)
Experiences of sharing personal health data for scientific research	0.092	0.056	2.748	.01	1.097 (0.983-1.223)
Constant	-0.762	0.295	6.676	.01	0.467 (0.262-0.832)

### Reliability and Validity of Measurement Instrument

#### Overview

The mean values of the 33-item scale range from 2.039 to 4.125, indicating that respondents generally showed a more positive attitude, and the SD ranges from 0.742 to 1.048, showing a reasonable distribution of the data. In this study, the kurtosis ranged from –0.436 to 1.116 and the skewness ranged from –0.949 to 0.659. An absolute skewness of less than 3 and a kurtosis of less than 10 indicate that the data generally follow a normal distribution (Multimedia Appendix 2).

#### Reliability of Measurement Instrument

The Cronbach coefficients ranged from 0.735 to 0.884 (privacy concern, α=.790; information control, α=.851; perceived risk, α=.735; trust, α=.782; perceived benefit, α=.884; moral motive, α=.840; perceived effectiveness of government regulation, α=.847; monetary benefit, α=.773; and sharing willingness, α=.783), all of which exceeded the cutoff value of 0.7, indicating internal consistency [32,33]. The scale items were further screened and analyzed by calculating item-scale correlation coefficient, CITC, and “Cronbach α if item was deleted” to identify the degree of homogeneity between the items and the overall scale (Table 5). For the “Cronbach α if item was deleted” condition, there was a significantly smaller value than the Cronbach α, value indicating that the item should not be deleted. Item-scale correlation coefficient and CITC values were all greater than 0.4 and significantly different (Table 5), indicating that the measurement instrument has good reliability and can be analyzed in the next step [34,35].

**Table 5:** Results of reliability analysis of measurement instrument.

Constructs and items			Item-scale correlation^a^		Corrected item-to-total correlation^a^		Cronbach α if item was deleted^b^		Cronbach α	
**PC^c^**										.790
	PC1	0.435		0.592		.744				
	PC2	0.513		0.583		.746				
	PC3	0.419		0.605		.739				
	PC4	0.568		0.556		.756				
	PC5	0.502		0.512		.768				
**IC^d^**										.851
	IC1	0.582		0.702		.806				
	IC2	0.666		0.697		.808				
	IC3	0.556		0.673		.818				
	IC4	0.654		0.692		.811				
**PR^e^**										.735
	PR1	0.400		0.547		.663				
	PR2	0.475		0.519		.694				
	PR3	0.452		0.612		.582				
**TR^f^**										.782
	TR1	0.640		0.664		.658				
	TR2	0.673		0.629		.696				
	TR3	0.709		0.571		.761				
**PB^g^**										.884
	PB1	0.719		0.769		.848				
	PB2	0.694		0.695		.866				
	PB3	0.712		0.723		.859				
	PB4	0.730		0.735		.856				
	PB5	0.720		0.687		.868				
**MB^h^**										.840
	MB1	0.401		0.714		.779				
	MB2	0.428		0.672		.798				
	MB3	0.482		0.606		.826				
	MB4	0.399		0.704		.785				
**PEGR^i^**										.847
	PEGR1	0.659		0.688		.811				
	PEGR2	0.588		0.714		.786				
	PEGR3	0.594		0.743		.758				
**MM^j^**										.773
	MM1	0.644		0.658		.642				
	MM2	0.551		0.557		.760				
	MM3	0.722		0.619		.684				
**SW^k^**										.783
	SW1	0.639		0.649		.677				
	SW2	0.693		0.590		.739				
	SW3	0.643		0.628		.701				

^a^Item-scale correlation coefficient and corrected item-to-total correlation were all greater than 0.4 and significantly different.

^b^Cronbach α if item was deleted was significantly smaller than the Cronbach α value.

^c^PC: privacy concern.

^d^IC: Information control.

^e^PR: perceived risk.

^f^TR: trust.

^g^PB: perceived benefit.

^h^MB: monetary benefit.

^i^PEGR: perceived effectiveness of government regulation.

^j^MM: moral motive.

^k^SW: sharing willingness.

#### Validity of Measurement Instrument

For each scale, the Kaiser-Meyer-Olkin value was 0.941, above the recommended value of 0.6, and the Bartlett test of sphericity reached statistical significance (*χ*^2^=30,237.196, *P*<.001), which demonstrated that the data collected from the questionnaire were well suited for a principal factor analysis [36]. An exploratory factor analysis was performed, which showed that all items had a communality value of greater than 0.4, indicating that the questionnaire information could be effectively analyzed. To determine whether the correspondence between constructs and items was consistent with the research hypothesis, factor rotation was conducted according to the maximum variance method based on the criteria of characteristic roots greater than 1. If it is basically consistent, the validity is good; if it is seriously inconsistent with the hypothesis or if the commonality value of an item is lower than 0.4, the validity is poor and the item may be considered for deletion (Multimedia Appendix 3).

Next, confirmatory factor analysis was used to test convergent validity and discriminant validity. The factor loading coefficients indicate the correlation between the constructs and items. If it is between 0.50 and 0.95, then it means that it is acceptable; otherwise it means that this item needs to be deleted and reanalyzed (Multimedia Appendix 4). Table 6 presents the results of the convergent validity and discriminant validity tests. Convergent validity can be tested by examining the standardized factor loading, composite reliability, and average variance extracted (AVE). As a result of this study, all reported AVE was greater than 0.5 and the composite reliability and standardized factor loading exceeded the threshold of 0.7 [36]. These measures indicate that the convergent validity of the measurement model was acceptable. Furthermore, a test of discriminant validity was conducted using Fornell-Larcker criteria as well as the heterotrait-to-monotrait ratio. As shown in Table 6, the main diagonal elements in *italics* denote the square roots of the AVEs, and the off-diagonal values represent the correlation coefficients between the constructs. All the diagonal values are greater than 0.7 and exceed the correlations between any pair of constructs [37]. Table 7 shows that all the heterotrait-to-monotrait ratio values are below the strict threshold of 0.85, which allows the establishment of discriminant validity [38]. Hence, this measurement instrument achieved adequate convergent validity and discriminant validity.

**Table 6:** Convergent validity and discriminant validity of constructs (Fornell-Larcker criterion).

Construct	PR^a^	PB^b^	SW^c^	IC^d^	PC^e^	MB^f^	MM^g^	PEGR^h^	TR^i^	AVE^j^	CR^k^
PR	*0.770* ^l^	—^m^	—	—	—	—	—	—	—	0.592	0.813
PB	–0.317	*0.783*	—	—	—	—	—	—	—	0.612	0.887
SW	–0.130	0.679	*0.769*	—	—	—	—	—	—	0.591	0.742
IC	–0.565	0.628	0.379	*0.768*	—	—	—	—	—	0.590	0.812
PC	0.440	–0.459	–0.266	–0.531	*0.786*	—	—	—	—	0.618	0.762
MB	–0.126	0.345	0.402	0.090	–0.138	*0.760*	—	—	—	0.578	0.844
MM	–0.220	0.705	0.690	0.430	–0.344	0.420	*0.783*	—	—	0.612	0.759
PEGR	–0.102	0.576	0.540	0.366	–0.300	0.366	0.531	*0.808*	—	0.654	0.850
TR	–0.316	0.657	0.492	0.597	–0.414	0.173	0.488	0.596	*0.783*	0.613	0.760

^a^PR: perceived risk.

^b^PB: perceived benefit.

^c^SW: sharing willingness.

^d^IC: information control.

^e^PC: privacy concern.

^f^MB: monetary benefit.

^g^MM: moral motive.

^h^PEGR: perceived effectiveness of government regulation.

^i^TR: trust.

^j^AVE: average variance extracted.

^k^CR: composite reliability.

^l^The diagonal *italicized* numbers are the square root of the average variance extracted for each construct.

^m^Not applicable.

**Table 7:** Discriminant validity of constructs (heterotrait-to-monotrait ratio).

Construct	PR^a^	PB^b^	SW^c^	IC^d^	PC^e^	MB^f^	MM^g^	PEGR^h^	TR^i^
PR	—^j^	—	—	—	—	—	—	—	—
PB	–0.371	—	—	—	—	—	—	—	—
SW	–0.166	0.840	—	—	—	—	—	—	—
IC	–0.694	0.740	0.491	—	—	—	—	—	—
PC	0.563	–0.565	–0.357	–0.681	—	—	—	—	—
MB	–0.155	0.401	0.508	0.111	–0.174	–	—	—	—
MM	–0.280	0.743	0.832	0.550	–0.456	0.527	—	—	—
PEGR	–0.123	0.666	0.687	0.444	–0.378	0.432	0.667	—	—
TR	–0.403	0.800	0.659	0.762	–0.550	0.217	0.644	0.743	—

^a^PR: perceived risk.

^b^PB: perceived benefit.

^c^SW: sharing willingness.

^d^IC: information control.

^e^PC: privacy concern.

^f^MB: monetary benefit.

^g^MM: moral motive.

^h^PEGR: perceived effectiveness of government regulation.

^i^TR: trust.

^j^Not applicable.

### Model Validation

#### Model Modification

Structural equation modeling was used to evaluate the degree of fit of the model. In general, a model is considered acceptable if the chi-square/degree of freedom (*χ*^2^/*df*) is between 2 and 5; root-mean-square residual (RMR) is less than 0.05; root-mean-square error of approximation (RMSEA) is less than 0.08; and goodness-of-fit index (GFI), normed fit index (NFI), comparative fit index (CFI) are greater than 0.9 [34]. Based on the model fit indices (*χ*^2^/*df*=8.982, RMSEA=0.063, RMR=0.037, GFI=0.911, NFI=0.919, CFI=0.928), it was evident that the hypothesized model needed to be modified to be more accurately fitting. The paths that are unreasonable or do not reach the significant level were modified based on theoretical basis: 2 paths predicting *sharing willingness from perceived effectiveness of government regulation* and *moral motive* were removed, and 1 path predicting *perceived benefit* from *information control* was added. After several revisions of the model fit, the measurement model was derived to be a good fit, with all goodness-of-fit indices (ie, *χ*^2^/*df*=2.637, RMSEA=0.048, RMR=0.032, GFI=0.950, NFI=0.955, CFI=0.962) meeting their respective common acceptance levels. It has been demonstrated that the final model has improved significantly over the initial model, which would provide a more objective and comprehensive representation of the relationship between the theoretical model and the empirical data.

#### Hypothesis Testing

**Figure 2:**
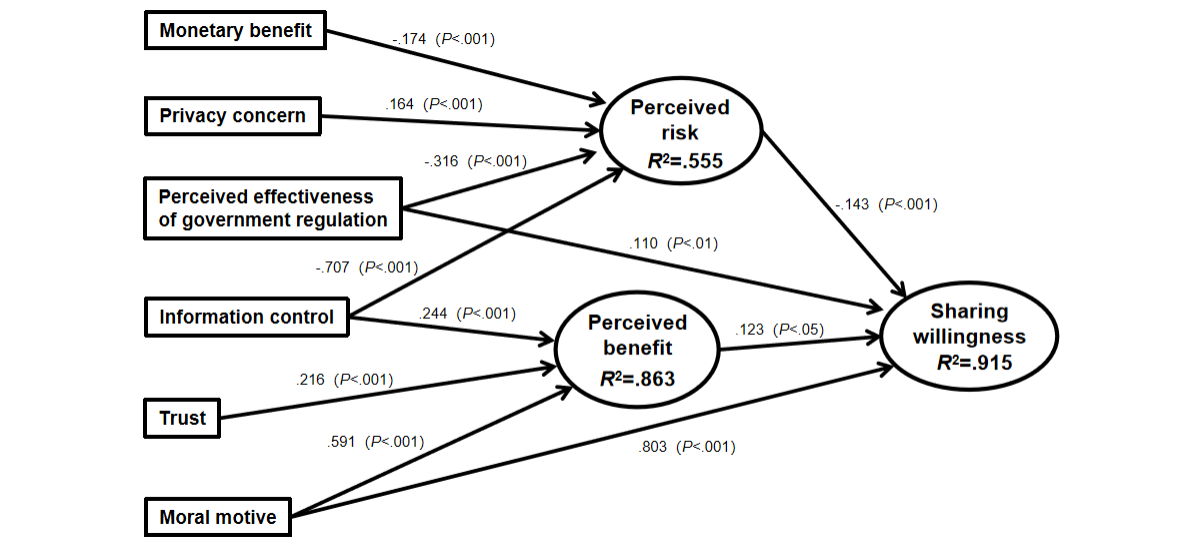
Results of the structural model.

The structural equation modeling analysis results provided empirical support for the proposed model. In the final model, all path coefficients between measured variables and factors were significant (2-tailed; *P*<.05). Table 8 clearly shows that moral motive (β=.803, *P*<.001), perceived risk (β=–.143, *P*<.001), perceived benefits (β=.123, *P*=.04), and perceived effectiveness of government regulation (β=.110, *P*=.001) were the main factors significantly affecting patients’ willingness to share personal health data in the maternal and child health hospitals, and moral motive seems to be the most powerful influencing factor (Figure 2 and Table 8). According to the model, 86.3% of the variance in perceived benefit, 55.5% in perceived risk, and 90.5% in sharing willingness can be explained by the entire model.

**Table 8:** Results of hypotheses testing.

Hypothesis	Path	Coefficient (β)	SE	*z* (critical ratio)	*P* value	Hypothesis validation
H1	PR^a^→SW^b^	–0.143	0.019	–6.294	<.001	Supported
H2	PB^c^→SW	0.123	0.063	1.962	.04	Supported
H3	PEGR^d^→SW	0.110	0.030	3.376	<.001	Supported
H4	PEGR→PR	–0.316	0.035	–10.172	<.001	Supported
H5	MM^e^→SW	0.803	0.061	11.894	<.001	Supported
H6	MM→PB	0.591	0.027	19.603	<.001	Supported
H7	MB^f^→PR	–0.174	0.028	–6.877	<.001	Supported
H8	IC^g^→PR	–0.707	0.043	–17.343	<.001	Supported
H9	IC→PB	0.244	0.023	9.298	<.001	Supported
H10	PC^h^→PR	0.164	0.035	4.858	<.001	Supported
H11	TR^i^→PB	0.216	0.033	6.457	<.001	Supported

^a^PR: perceived risk.

^b^SW: sharing willingness.

^c^PB: perceived benefit.

^d^PEGR: perceived effectiveness of government regulation.

^e^MM: moral motive.

^f^MB: monetary benefit.

^g^IC: information control.

^h^PC: privacy concern.

^i^TR: trust.

## Discussion

### Principal Findings

This empirical study identified key factors influencing patients’ willingness to share personal health data among patients in maternal and child health hospitals. A research framework was proposed by integrating the Theory of Planned Behavior and the Theory of Privacy Calculus, as well as corresponding measurement instruments. It has been found that moral motive, perceived benefit, and perceived effectiveness of government regulation have a significant positive effect on sharing willingness, and perceived risk has a significant negative effect. Health care data sharing is primarily motivated by altruistic motives, with moral motive having the strongest effect. In this study, patients who have experienced personal health information leakage and tertiary hospital visits are more likely to share their health information with health care providers. Other demographic characteristics, such as age, gender, education level, income, perceived health status, or place of residence, do not affect willingness to share health information.

### General Willingness to Share Personal Health Data

Medical services and health management have become increasingly dependent on information technology, and it has become a trend for patients to share their personal health information with health care providers via wearable devices and internet-based platforms. According to another previous study in China [39], 38.9% of the public would like to share their electronic health records (EHRs) with medical researchers, and 30% for other nonmedical purposes. These studies were conducted to demonstrate that most Chinese residents are cautious about sharing their EHRs with medical researchers. In another survey conducted by the China Youth Social Center, 88.8% of respondents reported that their data had been improperly processed [40]. Thus, it is likely that people who have used online health care services have different experiences and feelings than those who have not, with those who use it more frequently having higher privacy concerns and a stronger sense of privacy protection. In our study, patients who have not experienced personal health information leakage are more likely to share their health information. Similarly, Chinese researchers found that people who have never used online health care services are more likely to provide personal information to access these services [41].

The degree of informationization varies among different levels of medical institutions in China due to differences in construction expenditure and information technology capabilities. In general, tertiary hospitals should have higher policy requirements and a higher degree of information technology implementation than secondary hospitals and primary hospitals. Interconnecting health care data is impossible without the technical support of hospital informatization, and accordingly, different levels of informatization result in different patient experiences and levels of satisfaction. Therefore, the level of hospitals may be related to the willingness of patients to share information.

### Factors Affecting Willingness

As reported in a 2011 study, China had one of the highest percentages of users who frequently shared their personal information online for their own benefit [19]. Similar to other studies, this study found that participants were more likely to share their information if there was a societal benefit [42-44]. In addition, this study indicates that perceived risks outweigh perceived benefits, patients have a strong concern about data sharing, and are aware of privacy protection concerns. Several previous studies have reported consistent findings that privacy and security are important considerations for the public prior to sharing health information [45-48]. According to a survey of 932 Chinese residents, the participants were not inclined to share EHRs. Over one-half of the sample expressed negative attitudes toward the sharing of personal health information, suggesting that individuals are becoming increasingly aware of the importance of maintaining their personal information. Employment in the health care sector, experience with EHRs, understanding of the benefits of sharing health data, and awareness of potential risks all affect patients’ willingness to share their EHR data [9].

As indicated in the Theory of Privacy Calculus, when participants are aware of the explicit benefits of an activity, their perception of the associated risks decreases, and as a result, their behavior changes. It has been observed that people’s privacy decisions are inconsistent and that they might behave differently depending on the actual circumstances versus hypothetical situations [49]. Although accumulating evidence indicates that the perception of risk has a significant impact on willingness to share personal information [50,51], some studies have also shown an insignificant relationship between perceived risk and willingness to provide personal information [52]. The differences in perceived risk may be attributed to the particular results of this latent variable within the health care context, or the fact that data sharing involves different types of information [53], or different kinds of respondents, or they may also be explained by the fact that perceived risk and willingness to share are mediated by other variables [1] that need to be investigated further in-depth in the future. There is a wide variation in the willingness of people to share personal health data; however, researchers should focus on communicating their data practices effectively to minimize concerns about misuse of data and increase public trust [54].

As technologies used for information exchange advance, patients are concerned about organizations disclosing, transferring, and selling their personal information. Government legislation is considered to be one of the most common and fundamental methods of protecting personal data [55,56]. The privacy policy statements describe how health care organizations collect, manage, use, and disseminate patient health information. As supported by another study [1], the hypothesis that perceived effectiveness of government regulation has a positive impact on sharing willingness and a negative impact on perceived risk is supported in this empirical study. In recent years, the Chinese government has devoted considerable attention to the application and development of health care data, even though data sharing is not a government-controlled information technology in China. Furthermore, the health information infrastructure platform has now been enhanced in China, while population health information standards have been revised and security and protection systems have been improved. Several countries have also implemented policies and regulations relating to the protection of personal information, but not all citizens of those countries are confident that their government regulations will provide sufficient protection [57]. Although a detailed description of HIE technology was provided to ensure that respondents understood the context and purpose of data sharing, this does not guarantee that patients have read the privacy policy carefully, even if they have had relevant experience [58]. It has been demonstrated in some studies that perceived transparency of the privacy policy can reduce uncertainty associated with information-sharing processes, increase patient awareness of HIE, and provide knowledge about how it is carried out [59]. It is therefore necessary to promote transparency and public awareness about the policies governing data privacy protection, as well as reducing public concerns concerning the leakage of sensitive health data.

### Implications

The findings of this study contribute to completing our understanding of the traditional privacy calculus model beyond its 2 constituent variables (ie, perceived benefit and perceived risk). To our knowledge, only a few previous studies have integrated the Theory of Planned Behavior with the Theory of Privacy Calculus as their research framework [5]. The validity of the theoretical model was confirmed via a cross-sectional field survey. Our research contributes to the development of both the theoretical model and literature in the health care domain, and further extends it by adding several relevant constructs.

The findings of this study have several practical implications for policy makers and health care providers. A management recommendation was made based on the results regarding the facilitation of the continuous promotion of health care data sharing, the implementation of a coordinated regional development strategy, and the integration of health services. Health care data are usually generated by medical and health service organizations, public health institutions, and other subjects (eg, medical examination centers and insurance institutions). However, in China the aforesaid health care providers generally lack professional data analysis capabilities. As a result of privacy concerns, health care organizations and patients are cautious about sharing and utilizing health care data. Some individuals with the right to control data resources engage in illegal private sales or trades, misuse of data, and other behaviors that pose a serious threat to national security and economic interests. First, medical institutions should publicize the benefits and usefulness of sharing health information; improve patients’ perceptions of benefits; establish a subjective norm of mutual trust, respect, and mutual benefit; and encourage patients to share their health information more actively. Second, it is imperative to strengthen the technical skills of relevant technicians and to enhance communication on information technology security of personal health data in order to reduce perceived risks of misuse when it comes to protecting their personal data from loss as well as the persons who are allowed to access their data. Third, individual private information, including date type, data purpose, and demographics (eg, names, ID numbers, correspondence, occupation, household income) should be deidentified to enhance the trust of patients in health care organizations. A data sharing policy is designed to ensure that patients are informed about the use of their information [51]. Patients must consent to the sharing of their data, and the policy should ensure that all information is shared transparently and completely. Fourth, it is possible for hospitals to establish a department that is responsible for regularly educating patients about the use of their private health information, handling complaints regarding privacy breaches, and providing responses. The platforms of various hospitals are largely constructed independently and have mixed quality because there are no unified standards for health information sharing. Additionally, governments should promote laws and regulations regarding data privacy, clarify privacy data definitions, and define users’ rights and responsibilities. The characteristics of users should be considered, and targeted measures should be adopted to raise awareness of the privacy protection of EHRs, to develop technical skills and increase public awareness, and to create an environment conducive to privacy protection.

### Limitations

This study has several limitations that should be addressed. First, there are regional differences in the willingness to share personal health data. Medical resources in China are unevenly distributed among different regions, and economically developed regions have more financial, platform, and management advantages in promoting information sharing. The Yangtze River Delta represents the highest level of regional health information interconnection in China. However, generalizability may be limited because our sample consists of patients from a particular region. Second, the study selected some variables that may affect willingness to share based on the literature, focusing primarily on relevant factors at the individual level. While it also improved the influencing factors at the organizational level, such as perceived effectiveness of government regulation, other variables at the social, ethical, and technological levels were not considered. The underlying influence mechanism that integrates the Theory of Planned Behavior and the Theory of Privacy Calculus also needs to be further developed. Third, a self-report of willingness to share was assessed in this study, therefore there may have been a social desirability bias occurring. A privacy paradox suggests that individuals’ intentions to share information are not directly related to their actual behavior, and that other factors must be recognized as mediators [60-62]. Future studies may use a factorial design to explore mediators between intention and behavior gaps and how these affect perceptions of sharing personal health information.

To further generalize this framework, future work may expand the object, content, and scope of the empirical study. In addition, (1) research will be conducted in remote and grassroots areas and compared with advanced medical information technology regions. (2) There are still some potential factors that have not yet been included but are relevant to this theoretical model. There would be an opportunity for investigators to experiment in the future by varying the details provided regarding the risks and benefits associated with personal health data sharing, in addition to learning from experiences in other areas where data sharing is significant. (3) This study examined the factors influencing the willingness to share personal health data among patients in the maternal and child hospital, and further validation from the perspective of different stakeholders and application scenarios may be considered to compare the differences. (4) In addition, further studies should examine related issues such as the types and scope of health data that should be shared, forms of informed consent to share health data, and privacy protection measures. As a decision tool, privacy impact assessments could assist hospitals and government agencies in identifying and reducing the privacy risks associated with information systems.

### Conclusion

This paper contributes to expand the literature on patient privacy concerns and willingness to share personal health data by integrating the Theory of Planned Behavior and the Theory of Privacy Calculus. Patients with no prior experience with personal information disclosure and tertiary hospital visits were more likely to share their health data. Sharing willingness can be positively affected by perceived benefit, perceived risk, moral motives, and perceived effectiveness of government regulation. In particular, Chinese patients are motivated primarily by moral motives to improve public health and contribute to the diagnosis and treatment of diseases. By weighing the risks and benefits, the balance between data application and privacy security must be considered. It is imperative that relevant departments promote the awareness and transparency of health care data, laws, and regulations; clarify ownership of data; develop advanced technologies for privacy protection; promote the use of data sharing in scientific research, clinical practice, and health management; create public confidence in data sharing; and stimulate their motivation to share data.

## Appendix

Multimedia Appendix 1 STROBE (Strengthening the Reporting of Observational Studies in Epidemiology) statement—checklist of items that should be included in reports of cross-sectional studies.

Multimedia Appendix 2 Descriptive analysis of measurement instrument.

Multimedia Appendix 3 The structure matrix for exploratory factor analysis of measurement instruments.

Multimedia Appendix 4 Confirmatory factor analysis loading coefficients for measurement instruments.

## Abbreviations

AVE: average variance extracted
CFI: comparative fit index
CITC: corrected-item total correlation
EHR: electronic health record
GFI: goodness-of-fit index
HIE: health information exchange
NFI: normed fit index
RMR: root-mean-square residual
RMSEA: root-mean-square error of approximation
STROBE: Strengthening the Reporting of Observational Studies in Epidemiology

## Data Availability

The data sets from the questionnaires generated and analyzed during this study are not publicly available due ethical concerns but are available from the corresponding author on reasonable request.
