# Supplementary material for: Factors Influencing the Sharing of Personal Health Data Based on the Integrated Theory of Privacy Calculus and Theory of Planned Behaviors Framework: Results of a Cross-Sectional Study of Chinese Patients in the Yangtze River Delta
Source: J Med Internet Res. 2023 Jul 6;25:e46562. doi: 10.2196/46562 (PMC10359915; doi:10.2196/46562)
Supplement: Multimedia Appendix 2 [file jmir_v25i1e46562_app2.docx]

Appendix 2. Descriptive analysis of measurement instrument

| Constructs and items | Wording | | Mean±SD | Kurtosis | Skewness | Coefficient  of variation |
| --- | --- | --- | --- | --- | --- | --- |
| **Information Control (IC)** |  | |  |  |  |  |
|  | I have the right to decide who can use my health information | | 4.125±0.868 | 0.980 | -0.949 | 21.033% |
|  | It is up to me to decide what health information the hospital may share about me | | 3.898±0.840 | 0.479 | -0.594 | 21.539% |
|  | I can decide how the hospital will use my health information | | 3.943±0.845 | 0.578 | -0.655 | 21.438% |
|  | I have the right to decide what health information to provide to a hospital | | 3.974±0.796 | 0.440 | -0.563 | 20.024% |
| **Trust (TR)** |  | |  |  |  |  |
|  | I believe the hospital is trustworthy | | 3.795±0.779 | 0.568 | -0.397 | 20.528% |
|  | I believe that the health information provided to me by the hospital is true and reliable | | 3.876±0.768 | 0.615 | -0.477 | 19.811% |
|  | I believe hospitals can share information in a secure way | | 3.638±0.807 | 0.700 | -0.429 | 22.191% |
| **Perceived Benefit (PB)** |  | |  |  |  |  |
|  | Sharing health information can save you time during medical visits and assist you in finding the health information you require quickly | | 3.756±0.798 | 0.842 | -0.583 | 21.240% |
|  | Sharing health information can prevent patients from having to repeat tests | | 3.865±0.823 | 0.594 | -0.584 | 21.286% |
|  | Sharing health information helps validate the accuracy of previous scientific findings | | 3.651±0.805 | 0.756 | -0.493 | 22.043% |
|  | Sharing health information facilitates scientific research that is beneficial to the public | | 3.685±0.806 | 0.468 | -0.385 | 21.883% |
|  | Health information sharing facilitates patient care in hospitals | | 3.787±0.742 | 1.081 | -0.557 | 19.597% |
| **Monetary Benefit (MB)** |  | | | | | |
|  | I am willing to share my personal health data because I can get a good return | | 2.819±1.048 | -0.436 | 0.078 | 37.189% |
|  | Financial compensation is an important incentive for me to provide personal health data for scientific research | | 3.037±0.995 | -0.229 | -0.173 | 32.758% |
|  | Sharing personal health data (for scientific research) is a great way to subsidize revenue | | 3.114±0.963 | -0.080 | -0.116 | 30.918% |
|  | Sharing personal health data allows me to benefit financially from what I have | | 2.890±0.976 | -0.071 | 0.014 | 33.752% |
| **Perceived Risk (PR)** |  | | | | | |
|  | Providing my personal health data to the hospital could be potentially costly | | 2.489±0.881 | 0.011 | 0.236 | 35.413% |
|  | Providing my personal health data to a hospital can create many unforeseen problems | | 2.520±0.815 | 0.215 | 0.102 | 32.353% |
|  | Providing my personal health data to the hospital is risky | | 2.536±0.869 | 0.105 | 0.186 | 34.276% |
| **Privacy Concern (PC)** | |  | | | | |
|  | I am concerned that the personal health data I provide to the hospital may be misappropriated | | 2.232±0.935 | 0.425 | 0.659 | 41.875% |
|  | I'm worried that other people will find my personal health data online | | 2.202±0.877 | 0.426 | 0.548 | 39.817% |
|  | I am concerned that the personal health data I provide to the hospital will be used by others for other purposes | | 2.282±0.912 | -0.040 | 0.422 | 39.968% |
|  | Privacy protection of personal health data is very important in my mind compared to other things | | 2.039±0.777 | 0.368 | 0.474 | 38.086% |
|  | I am more sensitive to the way hospitals handle my personal health data than others (such as friends, relatives and colleagues) | | 2.355±0.804 | 0.031 | 0.139 | 34.132% |
| **Perceived Effectiveness of Government Regulation (PEGR)** | | | | | | |
|  | I believe the government will impose penalties on entities or individuals who violate privacy protection laws and regulations | | 3.683±0.832 | 0.567 | -0.455 | 22.604% |
|  | I believe that the government will defend my rights once it is discovered that my personal health data has been stolen | | 3.627±0.896 | 0.327 | -0.426 | 24.700% |
|  | I believe the government is capable of dealing with infringement issues resulting from the sharing of personal health data | | 3.507±0.904 | 0.327 | -0.426 | 25.780% |
| **Sharing Willingness (SW)** | | | | | | |
|  | I am willing to provide my personal health data to the hospital | | 3.566±0.816 | 0.775 | -0.455 | 22.874% |
|  | I am willing to share personal health data with hospitals to get better treatment | | 3.689±0.819 | 0.767 | -0.552 | 22.206% |
|  | I consent to the use of my personal health data by a doctor or researcher | | 3.425±0.898 | 0.123 | -0.336 | 26.214% |
| **Moral Motive (MM)** | | | | | | |
|  | Sharing personal health data because I want to help others | | 3.475±0.829 | 0.285 | -0.209 | 23.847% |
|  | Sharing personal health data is a matter of high moral character | | 3.220±0.935 | 0.177 | -0.224 | 29.034% |
|  | Sharing personal health data allows me to do something meaningful | | 3.614±0.821 | 0.470 | -0.356 | 22.717% |
