# Supplementary material for: Factors Influencing the Sharing of Personal Health Data Based on the Integrated Theory of Privacy Calculus and Theory of Planned Behaviors Framework: Results of a Cross-Sectional Study of Chinese Patients in the Yangtze River Delta
Source: J Med Internet Res. 2023 Jul 6;25:e46562. doi: 10.2196/46562 (PMC10359915; doi:10.2196/46562)
Supplement: Multimedia Appendix 3 [file jmir_v25i1e46562_app3.docx]

Appendix 3. The Structure Matrix for Exploratory Factor Analysis of Measurement Instruments

| Item | Cross-factor loading | | | | | | | | | Communality |
| --- | --- | --- | --- | --- | --- | --- | --- | --- | --- | --- |
|  | Factor 1 | Factor 2 | Factor 3 | Factor 4 | Factor 5 | Factor 6 | Factor 7 | Factor8 | Factor9 |  |
| PC1 | -0.155 | 0.033 | -0.028 | **0.806** | -0.025 | -0.239 | -0.098 | 0.030 | -0.085 | 0.751 |
| PC3 | -0.092 | -0.042 | 0.008 | **0.820** | -0.055 | -0.134 | -0.153 | -0.070 | 0.023 | 0.733 |
| PR1 | -0.005 | -0.128 | -0.007 | **0.824** | -0.040 | -0.084 | -0.142 | -0.114 | 0.038 | 0.739 |
| PC4 | -0.226 | 0.039 | -0.112 | 0.199 | -0.057 | -0.240 | **-0.786** | -0.148 | -0.034 | 0.806 |
| PC5 | -0.057 | -0.130 | -0.081 | 0.258 | -0.105 | -0.103 | **-0.829** | -0.047 | -0.019 | 0.804 |
| IC1 | 0.232 | -0.093 | 0.110 | -0.397 | 0.076 | **0.620** | 0.159 | 0.243 | 0.100 | 0.716 |
| IC2 | 0.397 | 0.037 | 0.224 | -0.159 | 0.131 | **0.676** | 0.257 | 0.017 | 0.005 | 0.775 |
| IC3 | 0.135 | 0.019 | 0.037 | -0.377 | 0.123 | **0.710** | 0.127 | 0.267 | 0.035 | 0.770 |
| TR1 | 0.252 | 0.042 | 0.403 | -0.078 | 0.156 | 0.161 | 0.079 | **0.696** | 0.081 | 0.781 |
| TR2 | 0.233 | -0.009 | 0.248 | -0.181 | 0.151 | 0.354 | 0.224 | **0.635** | 0.138 | 0.768 |
| MB1 | 0.060 | **0.831** | 0.148 | -0.019 | 0.095 | -0.023 | -0.039 | 0.028 | 0.092 | 0.737 |
| MB2 | 0.043 | **0.813** | 0.107 | -0.036 | 0.103 | 0.061 | 0.092 | -0.018 | 0.079 | 0.704 |
| MB3 | 0.380 | **0.688** | 0.126 | -0.084 | 0.095 | -0.145 | 0.003 | 0.190 | -0.156 | 0.731 |
| MB4 | 0.029 | **0.839** | 0.059 | -0.027 | 0.134 | 0.043 | 0.051 | -0.057 | 0.088 | 0.742 |
| PEGR1 | 0.179 | 0.078 | **0.722** | 0.006 | 0.371 | 0.074 | 0.182 | 0.141 | 0.139 | 0.775 |
| PEGR2 | 0.185 | 0.198 | **0.819** | -0.061 | 0.026 | 0.101 | 0.040 | 0.201 | 0.064 | 0.805 |
| PEGR3 | 0.195 | 0.191 | **0.823** | 0.028 | 0.151 | 0.091 | 0.067 | 0.106 | 0.085 | 0.806 |
| PB1 | **0.728** | 0.102 | 0.210 | -0.056 | 0.205 | 0.272 | 0.159 | 0.060 | 0.199 | 0.771 |
| PB2 | **0.637** | 0.031 | 0.179 | -0.154 | 0.135 | 0.208 | 0.133 | 0.433 | 0.093 | 0.738 |
| PB3 | **0.708** | 0.225 | 0.219 | -0.173 | 0.227 | 0.083 | 0.053 | 0.225 | 0.075 | 0.747 |
| PB4 | **0.647** | 0.164 | 0.161 | -0.116 | 0.390 | 0.329 | 0.073 | 0.027 | 0.099 | 0.761 |
| PB5 | **0.542** | 0.043 | 0.231 | -0.040 | 0.325 | 0.091 | 0.404 | 0.133 | 0.276 | 0.722 |
| SW1 | 0.353 | 0.186 | 0.269 | -0.002 | 0.239 | 0.079 | 0.071 | 0.139 | **0.739** | 0.866 |
| SW3 | 0.272 | 0.239 | 0.141 | -0.040 | 0.593 | 0.073 | 0.024 | 0.194 | **0.439** | 0.741 |
| MM1 | 0.260 | 0.216 | 0.206 | -0.080 | **0.803** | 0.075 | 0.106 | 0.060 | -0.026 | 0.830 |
| MM3 | 0.343 | 0.192 | 0.193 | -0.070 | **0.651** | 0.187 | 0.126 | 0.143 | 0.217 | 0.739 |

Note: Extraction Method: Principal Component Analysis. Rotation Method: the maximum variance method. Major loadings for each item are bolded.
