# Supplementary material for: Factors Influencing the Sharing of Personal Health Data Based on the Integrated Theory of Privacy Calculus and Theory of Planned Behaviors Framework: Results of a Cross-Sectional Study of Chinese Patients in the Yangtze River Delta
Source: J Med Internet Res. 2023 Jul 6;25:e46562. doi: 10.2196/46562 (PMC10359915; doi:10.2196/46562)
Supplement: Multimedia Appendix 4 [file jmir_v25i1e46562_app4.docx]

Appendix 4. Confirmatory Factor Analysis Loading Coefficients for Measurement Instruments

| Construct | Item | Coefficient (β) | Std. Error | Critical ratio | *P value* | Std. Estimate |
| --- | --- | --- | --- | --- | --- | --- |
| Perceived Risk | PR1 | 1.000 | - | - | - | 0.781 |
|  | PR2 | 0.890 | 0.028 | 31.415 | <0.001 | 0.737 |
|  | PR3 | 0.982 | 0.030 | 33.163 | <0.001 | 0.788 |
| Perceived Benefit | PB1 | 1.000 | - | - | - | 0.757 |
|  | PB2 | 1.000 | 0.028 | 36.111 | <0.001 | 0.776 |
|  | PB3 | 1.041 | 0.027 | 38.008 | <0.001 | 0.811 |
|  | PB4 | 1.036 | 0.027 | 37.683 | <0.001 | 0.805 |
|  | PB5 | 0.905 | 0.026 | 35.283 | <0.001 | 0.760 |
| Sharing Willingness | SW1 | 1.000 | - | - | - | 0.743 |
|  | SW2 | 1.170 | 0.035 | 33.688 | <0.001 | 0.789 |
| Information Control | IC1 | 1.000 | - | - | - | 0.796 |
|  | IC2 | 0.935 | 0.026 | 35.877 | <0.001 | 0.765 |
|  | IC3 | 0.897 | 0.026 | 34.499 | <0.001 | 0.740 |
| Privacy Concern | PC1 | 1.000 | - | - | - | 0.880 |
|  | PC2 | 0.811 | 0.032 | 25.590 | <0.001 | 0.688 |
| Monetary Benefit | MB1 | 1.000 | - | - | - | 0.683 |
|  | MB2 | 1.279 | 0.042 | 30.569 | <0.001 | 0.801 |
|  | MB3 | 1.164 | 0.039 | 30.135 | <0.001 | 0.785 |
|  | MB4 | 1.139 | 0.039 | 29.291 | <0.001 | 0.757 |
| Moral Motive | MM1 | 1.000 | - | - | - | 0.830 |
|  | MM2 | 0.891 | 0.025 | 35.189 | <0.001 | 0.733 |
| Perceived Effectiveness of Government Regulation | PEGR1 | 1.000 | - | - | - | 0.789 |
|  | PEGR2 | 1.059 | 0.028 | 38.228 | <0.001 | 0.828 |
|  | PEGR3 | 0.948 | 0.025 | 37.309 | <0.001 | 0.807 |
| Trust | TR1 | 1.000 | - | - | - | 0.812 |
|  | TR2 | 0.943 | 0.027 | 34.459 | <0.001 | 0.754 |
